# Elucidating the Photophysics and Nonlinear Optical Properties of a Novel Azo Prototype for Possible Photonic Applications: A Quantum Chemical Analysis

**DOI:** 10.1021/acsomega.4c04240

**Published:** 2024-09-20

**Authors:** Sàvio Fonseca, Neidy S. S. dos Santos, Herbert C. Georg, Tertius L. Fonseca, Patricio F. Provasi, Kaline Coutinho, Sylvio Canuto, Antônio R. da Cunha, Rodrigo Gester

**Affiliations:** † Programa de Pós-Graduação em Química, Universidade Federal do Sul e Sudeste do Pará, Marabá, Pará 68507-590, Brazil; ‡ Instituto de Física, Universidade Federal de Goiás, Goiânia, Goiás 74690-900, Brazil; § Department of Physics, IMIT, Northeastern University, CONICET, Avenue Libertad 5500, Corrientes W 3404 AAS, Argentina; ∥ Instituto de Física, Universidade de São Paulo, Rua do Matão 1371, São Paulo, São Paulo 05588-090, Brazil; ⊥ Centro de Ciências de Balsas, Universidade Federal do Maranhão, Balsas, Maranhão 65800-000, Brazil; # Faculdade de Física, Universidade Federal do Sul e Sudeste do Pará, Marabá, Pará 68507-590, Brazil

## Abstract

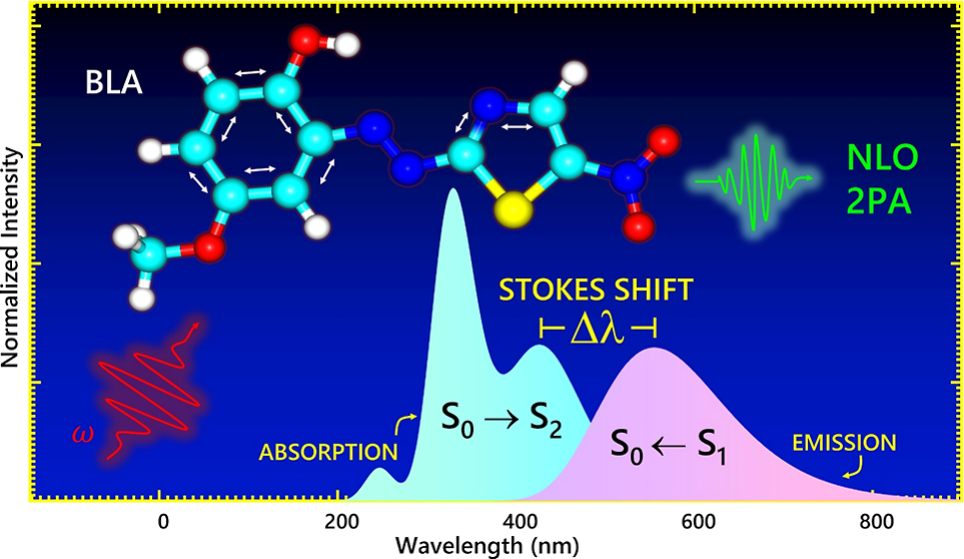

The photophysics and nonlinear optical responses of a novel nitrothiazol-methoxyphenol molecule were investigated using density functional theory (DFT) and time-dependent density functional theory (TD-DFT) methods with the polarizable continuum model to take the solvent effect into account. Special attention is paid to the description of the lowest absorption band, characterized as a strong π → π* state in the visible region of the spectrum. The TD-DFT emission spectrum analysis reveals a significant Stokes shift of more than 120 nm for the π → π* state in gas phase condition. The results show a great influence of the solvent polarity on the nonlinear optical (NLO) response of the molecule. Specifically, the second harmonic generation hyperpolarizability β(−2ω; ω, ω) shows a large variation from gas to aqueous solvent (82 × 10^–30^ to 162 × 10^–30^ esu), exhibiting notably higher values than those reported for standard compounds such as urea (0.34 × 10^–30^ esu) and *p*-nitroaniline (6.42 × 10^–30^ esu). Furthermore, a two-photon absorption analysis indicates a large cross-section (δ^2PA^ = 77 GM) with superior performance compared to several dyes. These results make the molecule quite interesting for nonlinear optics.

## 1 Introduction

Nonlinear optical (NLO) molecules and materials have been a continuous source of research for the past three decades, driven by their potential applications in optoelectronics, photonics, and communication technologies, such as optical data processing and storage.^1−4^ They are also attracting growing interest for possible applications in bioimaging, since the second harmonic generation (SHG) technique can produce high-resolution images from deep within biological tissues.^5−7^ Among the various NLO chromophores, SHG probes are typically designed by incorporating donor (D) and acceptor (A) groups, linked via a π-system, which provides the asymmetry required for quadratic NLO phenomena. These materials are not only synthetically accessible but they also have low dielectric constant and fast electronic responses, making them preferable to inorganic materials. Understanding, controlling and optimizing the NLO responses of organic compounds is fundamental, requiring experimental and quantum chemical characterizations to elucidate NLO structure–property relationships, including the influence of donor and acceptor forces and the nature of the π-conjugated segment.^8−17^

On the other hand, given that experimental property determination typically occurs in condensed phases, it is crucial to examine how environmental factors, such as the effects of solvents, impact NLO responses. Experimental studies have demonstrated that solvent effects can significantly alter the NLO response, as evidenced by compounds like *para*-nitroaniline (PNA), donor–acceptor polyenes, and thiophene chromophores.^18−23^ From these studies, it has been observed that more polar solvents tend to enhance the NLO responses. This highlights the need to take into account the effects of solvents in the design and application of NLO materials. Solvatochromism is an example of how a solvent can influence a specific molecular property. Solvatochromic effects arise from interactions between solute and solvent, leading to changes in the shape, intensity, and position of spectral lines.^23^ In this study, we utilized implicit schemes where the solvent is modeled as a polarizable continuum medium, characterized by its macroscopic properties, such as the dielectric constant.^24^

**Figure 1:**
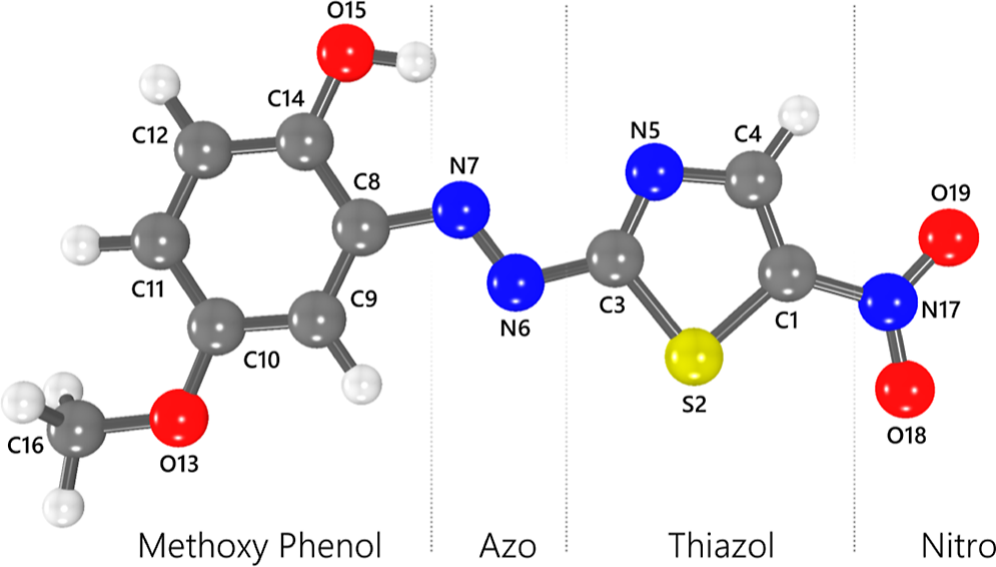
Molecular structure of the NTAMP molecule.

Quantum chemical studies on the physicochemical properties of NLO molecules provide complementary information to understand the relationships between their structural, electronic, and optical properties and therefore to design new molecules.^1^ In this context, 2-[2′-(5-nitrothiazolyl)azo]-4-methoxyphenol (NTAMP) depicted in Figure 1 emerges as a challenge.^25^ Despite being a potential prototype for novel devices, none of its optical properties have been investigated yet. Furthermore, even when the knowledge of the electronic structure of NTAMP has advanced, there is no information on how the environment influences these properties. It has been observed that this compound presents strong and broad excitation in the visible spectral region. However, contrary to expectations, this transition has been assigned as a weak state n → π*, which is puzzling, since these excitation types are not usually associated with strong intensities.

To better understand certain aspects of NTAMP photophysics, we drive a detailed investigation of its optical properties using theoretical quantum chemical methods. We particularly focus on the solvent and how its interactions can modulate the optical parameters of interest. Furthermore, we consider both the static field and the frequency-dependent light, which are crucial to understanding the effects of the SHG.^26^ Although they contradict the original assignment, all the used density functional theory (DFT) approaches have identified the three low-lying electronic bands observed previously experimentally and, instead, we find that it is a strong excitation π → π*. Additionally, despite the substantial values of the Stokes shift coefficients and NLO estimated for this azo compound, the solvent enhances these parameters, suggesting potential applications such as sensors, molecular probes, and optical devices such as sensors, and organic light-emitting diodes, or even solar cells.

## 2 Methodology

The NLO parameters of interest are the first hyperpolarizability (β) and the two-photon cross-section (δ). The first of them is a three rank tensor with 27 components. At static conditions or frequency-independent light, a representative quantity related to this tensor is given as^26^
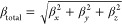
1where
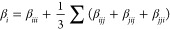
2

Another very important quantity for fotonics, related to the second-harmonic generation phenomenon, is the frequency-dependent first hyperpolarizability, β(−2ω; ω, ω).^26^ For frequency-dependent light, this optical coefficient can be obtained within the hyper-Rayleigh scattering (HRS) formalism as^27^
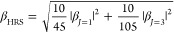
3

In this equation, β_*J*=1_ and β_*J*=3_ parameters correspond respectively to the dipolar and octupolar tensors, respectively written like
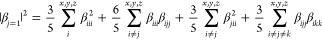
4and
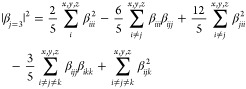
5

These two tensors can be combined to give the anisotropic factor ρ = |β_*J*=3_|/|β_*J*=1_|, defining the dipolar, Φ_*J*=1_ = 1/(1 + ρ), and octupolar, Φ_*J*=3_ = ρ/(1 + ρ) contributions to the first hyperpolarizability.

Calculations of the first hyperpolarizability were performed in Gaussian 16 code^28^ considering different DFT-based methods and the 6-311++G(d,p) basis set. Finally, the optical coefficients were posteriorly analyzed using the Multiwfn program.^29^ The same setup was applied to obtain the electronic excitations.

The effect of the solvent on the two-photon absorption mechanism (2PA) was also accounted for. It is a third-order phenomenon related to applications like photodynamic therapy, communication devices, telecommunications, integrated chips, and so on.^30^

Within the quantum mechanics formalism, 2PA is studied using a time-dependent perturbation theory. Considering a monochromatic linearly polarized light, the two-photon cross-section (δ_a.u._^2PA^) is given as a transition from |0⟩ to |*f*⟩ as
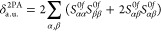
6

In this equation, the indices α and β over the three Cartesian coordinates, being *S*_αβ_^0*f*^ the αβ-th component of the two-photon tensor, which the mathematical form is given as
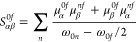
7where μ_*a*_^pq^ and o ω_*pq*_ are, respectively, the *a*-th component of the one-photon transition moment and the excitation energy for a transition going from |*p*⟩ to |*q*⟩. Note that δ_a.u._^2PA^ is in atomic units, but it can be transformed to GM (Göppert-Mayer) units by the following relation
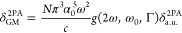
8

In this context, *N* is an integer that depends on the experimental setup, *a* is the fine structure constant, *a*_0_ is the Bohr radius, ω is the energy of the incoming photon, *c* is the speed of light, and *g*(2ω, ω_0_, Γ) is the line-shape function. All calculations for 2PA were carried out using the Dalton program^31^ and the 6-311++G(d,p) basis set.

All molecular geometries were obtained within the DFT framework^32−34^ using the ωB97XD method^35,36^ with the Pople 6-311++G(d,p) basis set.^37,38^ We have computed the geometry of the first excited state to investigate the emission excitation. In addition, the electronic properties considered here were also calculated using the M06-2X,^39^ and CAM-B3LYP^40^ methods. The solvent effects on the geometry and electronic properties were taken into account using the integral-equation version of the polarizable continuum model (PCM).^24,41,42^

The calculation of hyperpolarizability has consistently presented challenges, especially for compounds with donor and acceptor substituents due to the inherently nonlocal nature of their response. The CAM-B3LYP,^40^ M06-2X^39^ and ωB97X-D^35,36^ functionals belong to a new class of DFT functionals known as range-separated functionals, which effectively capture both short-range and long-range interactions.^43,44^

## 3 Results and Discussion

### 3.1 Geometry Analysis

The environment can affect diverse molecular properties, both directly, without appreciable modifications in the geometry of the solute, and indirectly, by inducing significant changes in the solute geometry. In particular, molecules with electron donor and acceptor moieties connected by a π-electron bridge may be largely affected by polar environments.^45,46^ In some cases, drastic geometric changes may occur upon solvation, leading the solute molecules to a cyanine-like state^47^ or even to a zwitterionic state.^45,46^ NLO properties may also suffer drastic changes as a result of both the direct and indirect solvent effects.^47^

Table 1 shows all the bond lengths of NTAMP and two bond length alternation (BLA) coordinates, selected to monitor the structure of the phenol and thiazol rings, respectively. The BLA coordinates, χ_P_ and χ_T_, are defined as
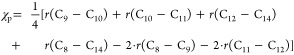
9
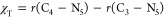
10

**Table 1:** Bond Length and Bond Length Alternation (BLA) Coordinates, in Å, Obtained for NTAMP in Gas Phase and Solvent for the Ground and First Excited States Calculated with TD-ωB97XD/6-311++G(d,p)

	ground state		emission state	
	gas phase	water	gas phase	water
Bond Lengths				
C_1_–S_2_	1.720	1.720	1.737	1.727
C_1_–C_4_	1.365	1.367	1.365	1.384
C_4_–N_5_	1.355	1.353	1.356	1.327
C_3_–N_5_	1.305	1.308	1.329	1.342
S_2_–C_3_	1.732	1.728	1.748	1.761
C_3_–N_6_	1.401	1.400	1.332	1.330
N_6_–N_7_	1.249	1.249	1.233	1.305
N_7_–C_8_	1.391	1.391	1.366	1.367
C_8_–C_9_	1.399	1.400	1.399	1.376
C_9_–C_10_	1.381	1.382	1.385	1.409
C_10_–C_11_	1.405	1.406	1.397	1.418
C_10_–O_13_	1.358	1.357	1.358	1.325
C_11_–C_12_	1.385	1.384	1.389	1.376
C_12_–C_14_	1.391	1.391	1.381	1.398
C_8_–C_14_	1.408	1.408	1.408	1.441
C_14_–O_15_	1.341	1.345	1.353	1.315
C_1_–N_17_	1.438	1.430	1.420	1.397
N_17_–O_18_	1.218	1.221	1.220	1.233
N_17_–O_19_	1.215	1.218	1.224	1.235
BLAs				
χ_P_	0.005	0.005	–0.001	0.040
χ_T_	0.050	0.045	0.027	–0.015

The χ_P_ BLA coordinate gives us information about the general state of the phenyl ring. As discussed in a previous paper,^47^ when χ_P_ is near zero, the ring is aromatic, while a value near 0.1 Å means that the ring is quinoidal, with the symmetry axis of the quinoidal ring being along the bonds C_14_–O_15_ and C_10_–O_13_. The χ_T_ BLA coordinate, in its turn, gives us information about the conjugation state of the N–C bonds in thiazol ring. A positive value of χ_T_, around 0.07 Å, means that C_4_–N_5_ is a single bond and C_3_–N_5_ is a double bond, as expected for the molecule in normal, nonzwitterionic state. A pronounced negative value of χ_T_ means that the N–C bonds in the thiazol ring are reversed, which means that the molecule is in a zwitterionic state.

Now, from Table 1 we observe that no significant change occurs in any bond length, in the ground state, upon solvation. Both in gas phase and in water, χ_P_ is very close to zero, which means an aromatic phenyl ring and χ_T_ is around 0.05 Å, which means a pronounced single–double conjugation in the N–C bonds in the thiazol ring. Also, the N_6_–N_7_ bond length is around 1.25 Å, which is very typical of a double bond. Therefore, these results indicate that NTAMP suffers no appreciable charge separation in the ground state upon solvation.

On the other hand, for the emission state, that is, the minimum energy geometry of the first excited state, the picture is quite different. Many bond lengths change appreciably upon solvation. In particular, χ_P_, which is essentially zero in gas phase, increases to 0.04 Å in water, showing that the phenyl ring changes to a structure that lies in the midway between aromatic and quinoidal. Accordingly, the bonds C_10_–O_13_ and C_14_–O_15_ also suffer a significant decrease, assuming a more double-bond character, which is in line with the more quinoidal structure of the phenyl ring. The other BLA coordinate, χ_T_, changes from 0.027 Å in gas phase to −0.015 Å in water, which means that the single–double conjugation in the N–C bonds in the thiazol ring are inverted. Also, the N_6_–N_7_ bond suffers a large increase from 1.233 to 1.305 Å, which is a significant change toward a single bond character.

These results show that, in the emission state, NTAMP undergoes a significant charge separation upon solvation. However, for characterizing a zwitterionic state, we would expect χ_P_ to be closer to 0.1 Å and χ_T_ closer to −0.07 Å. Also, the N_6_–N_7_ bond should be closer to 1.4 Å, that is a typical value for a single N–N bond. Therefore, we conclude that the molecule does not reach a zwitterionic state, but rather a cyanine-like state, which is in between the normal, non charge separated state and the zwitterionic state.

### 3.2 Electronic Transitions and Stokes Shift

Waheeb and collaborators have reported the electronic absorption spectra of the NTAMP molecule^25^ and identified a broad and intense band at 397 nm. The authors assigned this excitation as a n → π*, which is questionable, as this type of transition generally exhibits low intensity.

To elucidate the electronic transitions and have a better understanding of NTAMP’s photophysics, we have performed time-dependent density functional theory calculations with the molecule both in gas phase and in water. A summary of the results is presented in Table 2. The results for the absorption with the M06-2X functional show the presence of a S_0_ → S_1_ transition lying at 494.5 nm with an n → π* symmetry. This is confirmed by the results obtained with CAM-B3LYP and ωB97XD which also indicate that this transition has null oscillator strength, thus being a black state that does not appear in the absorption spectra. Consequently, this excitation is not meaningful, and therefore, omitted in Table 2.

**Table 2:** Lowest Absorption and Emission Bands, as Well as the Stokes Shifts Calculated for the NTAMP Molecule at Gas Phase and Aqueous Conditions

		absorption (π → π*)			emission (π ← π*)			Stokes Shift
method		λ_abs_ (nm)	Osc.	contribution	λ_em_ (nm)	Osc.	contribution	Δλ = λ_em_ – λ_abs_ (nm)
M06-2X	gas	(S_0_ → S_2_)428.9	0.319	H → L (93%)	(S_0_ ← S_1_)555.2	0.247	H ← L (96%)	126.3
	water	(S_0_ → S_2_) 462.0	0.621	H → L (90%)	(S_0_ ← S_1_) 651.3	0.699	H ← L (96%)	189.3
CAM-B3LYP	gas	(S_0_ → S_2_) 443.1	0.302	H → L (91%)	(S_0_ ← S_1_) 576.4	0.239	H ← L (95%)	124.3
	water	(S_0_ → S_2_) 479.1	0.594	H → L (87%)	(S_0_ ← S_1_) 677.4	0.680	H ← L (95%)	234.3
ωB97XD	gas	(S_0_ → S_2_) 434.3	0.321	H → L (88%)	(S_0_ ← S_1_) 560.1	0.256	H ← L (93%)	125.8
	water	(S_0_ → S_2_) 469.6	0.620	H → L (84%)	(S_0_ ← S_1_) 662.5	0.706	H ← L (93%)	192.9

**Figure 2:**
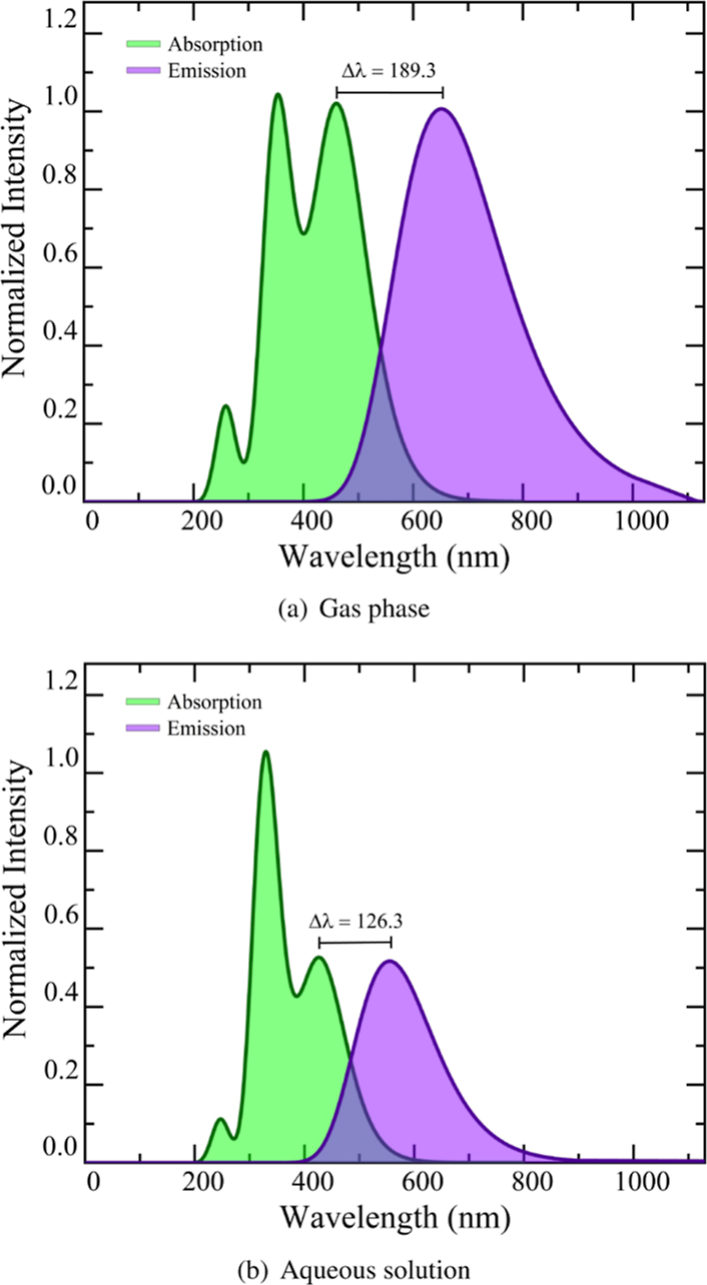
Electronic emission and absorption spectra calculated at the M06-2X/6-311++G(d,p) level for NTAMP in gas phase and aqueous solution.

**Figure 3:**
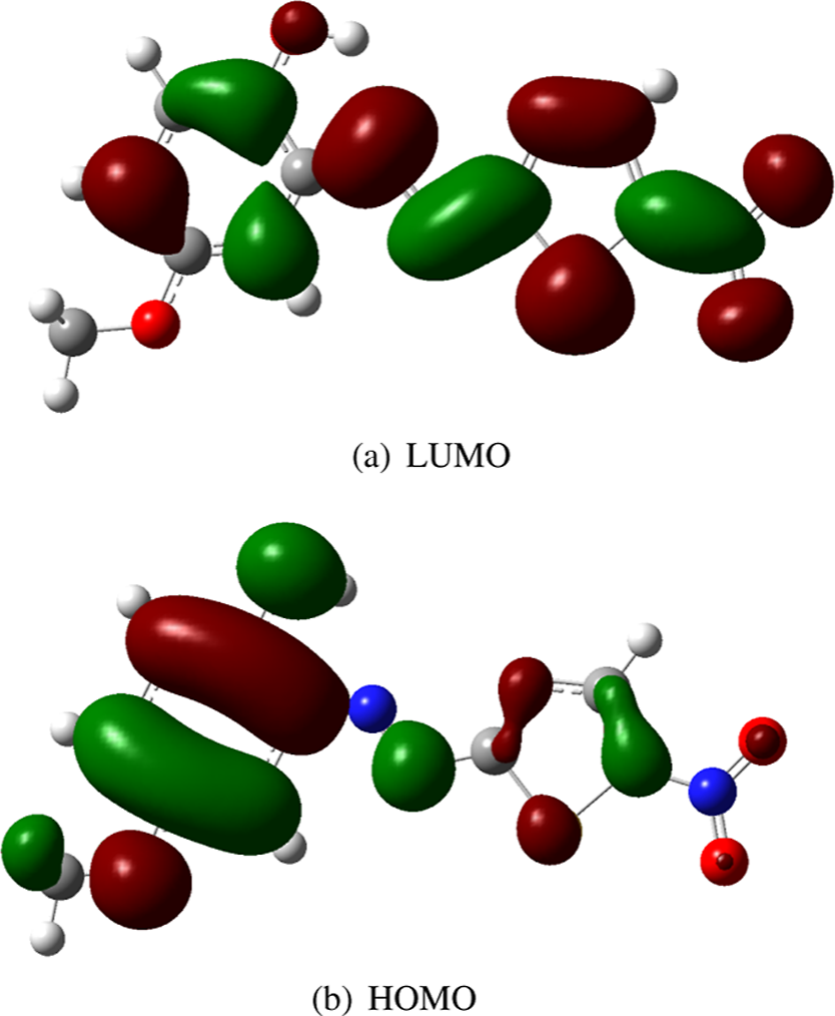
Kohn–Sham molecular orbitals involved in the lowest absorption and emission bands.

Additionally, Figure 2 illustrates the electronic absorption and emission spectra calculated in gas phase and aqueous solution. The band observed at 428.9 nm for the isolated molecule exhibits a strong oscillator strength of 0.319. However, after analyzing the molecular orbitals as depicted in Figure 3, it becomes clear that this excitation is not a n → π* transition as previously assigned, but rather a π → π* transition.

We obtained the minimum energy geometry in the lowest excited state and it corresponds to the same π → π* state. The emission then is calculated at 555.2 nm, suggesting a large Stokes shift, with Δλ = λ_em_ – λ_abs_ = 126.3 nm.

The solvent environment has a significant impact on the electronic excitations of NTAMP. The most noticeable effect comes from the intensities of the transitions. According to the experimental results obtained in methanol, the lowest absorption band should be the most intense, which is not observed in the gas phase spectrum shown in Figure 2a. However, including polarization effects using PCM helps to address this issue, as illustrated in Figure 2b.

In addition, the solvent moves this absorption band to 462 nm, a bathochromic shift of 33.1 nm compared to the gas phase molecule. The fluorescence also occurs at lower energies, specifically at 651.3 nm, resulting in a Stokes shift of 189.3 nm, indicating an improvement compared to the same Stokes shift in the gas phase condition.

### 3.3 Intramolecular Charge-Transfer

A pronounced intramolecular charge transfer (ICT) is likely to occur in molecules possessing a D-π–A structure, upon interaction with polar environments or following transitions to some excited states. And such charge transfer is quite relevant for understanding molecular solvatochromism and the effects in NLO properties.

As shown in Table 3, the ground state dipole moment in the gas phase is 6.5 D. However, following a vertical excitation, the dipole moment increases to 14.9 D, indicating a large polarization of the electronic charge.

**Table 3:** Ground State (GS) and Franck–Condon State (FC) Dipole Moments (μ/D)

	gas phase		water	
method	μ_GS_	μ_FC_	μ_GS_	μ_FC_
M06-2X	6.5	14.9	8.0	19.3
CAM-B3LYP	6.6	14.8	8.1	19.2
ωB97XD	6.5	14.1	8.0	18.2

**Figure 4:**
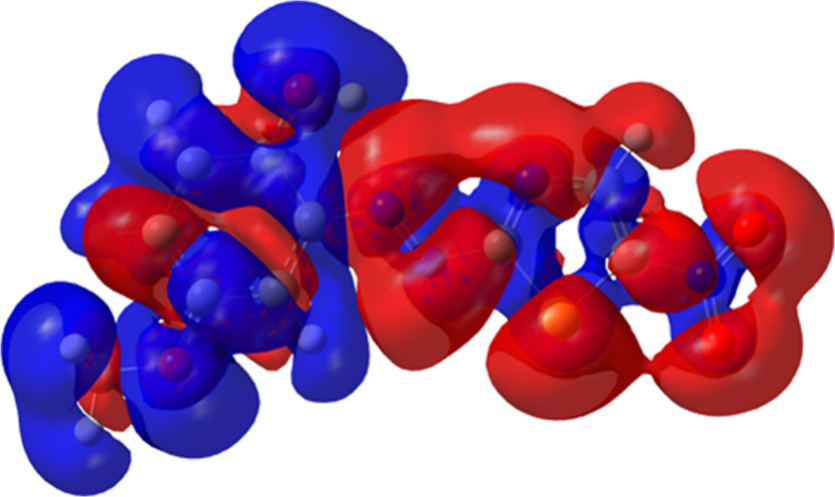
Electron density difference (Δρ) between the FC excited state and the ground state computed with TD-CAM-B3LYP/6-311++G(d,p) in gas phase. In red it shows the regions of density increase, while in blue the regions of density decrease. A visualization threshold of 0.0001 au was applied.

Figure 4 shows the electron density difference (Δρ) between the FC excited state and the ground state of the isolated molecule. We observe that there is a significant migration of electron density from the methoxy phenol moiety of the molecule to the other moiety, clearly characterizing the π → π* excited state as an ICT state.

In water, these effects are more pronounced. For example, the ground state dipole moment is now computed as 8.0 D, indicating a polarization, attributed to the solvent, of approximately 20%. In contrast, the Franck–Condon excited state exhibits a value of 19.3 D, approximately 39% greater than the gas phase value. Thus, the solvent further enhances the ICT phenomenon.

### 3.4 First Hyperpolarizability

Table 4 presents the results for the static (β_total_) and frequency-dependent (β_HRS_) first hyperpolarizabilities, enabling analysis of the chromophore’s performance. Static hyperpolarizabilities represent an inherent material response, independent of the incident radiation. Under gas phase conditions, a large value of 45 × 10^–30^ esu is obtained. For comparison, our DFT results for the first static hyperpolarizability are comparable to the second-order Møller–Plesset perturbation theory (MP2) results for azo-enaminone compounds of around 50 × 10^–30^ esu,^10^ where the azo group plays an important role in the nonlinear activities of the system. However, considering the presence of the solvent, this parameter is considerably increased to 133 × 10^–30^ esu, signifying a large polarization effect of 196% relative to the isolated molecule. This finding is consistent with previous studies showing that solvent effects can significantly increase the NLO response, as demonstrated for azochromophore with tricyanopyrrole acceptor moiety,^48^ twisted Möbius annulenes,^49^ organic biphotochrome,^50^ push–pull phenylpolyenes,^51^ pyridinium-*N*-phenoxide betaines^52^ and organic molecules.^53^ Additionally, it is noteworthy that NTAMP performs exceptionally well compared to other standard optical materials. For example, the first hyperpolarizabilities of urea and *p*-nitroaniline have been reported as 0.3 × 10^–30^ and 6.4 × 10^–30^ esu, respectively.^54,55^ Therefore, NTAMP appears to be a promising compound for NLO applications.

**Table 4:** First Hyperpolarizability (β/10^–30^ esu), Energy Gap (eV), Depolarization Ration (DR), Anisotropy Ratio (ρ), Dipolar (Φ_*J*=1_), and Octupolar (Φ_*J*=3_) contributions^a^^b^

	M06-2X		CAM-B3LYP		ωB97XD	
property	gas	water	gas	water	gas	water
β_total_	44.61	133.28	47.15	145.77	43.04	131.09
β_*x*_	–43.74	–130.26	46.23	–142.50	–42.13	–127.88
β_*y*_	87.78	28.27	9.24	30.71	8.78	28.84
β_*z*_	0.00	0.00	0.00	0.00	0.00	0.00
β_HRS_	68.00	127.51	81.85	162.93	68.01	129.62
DR	5.00	5.02	5.01	5.03	5.02	5.04
*E*_gap_	4.65	4.61	4.93	4.87	6.08	6.01
Φ_*J*=1_	0.551	0.552	0.551	0.552	0.552	0.553
Φ_*J*=3_	0.449	0.448	0.449	0.448	0.448	0.447
ρ	0.81	0.81	0.81	0.81	0.81	0.81

a The frequency-dependent results were calculated at λ = 1064 nm.

b The converting value for β are 1 au = 8.63922 × 10^–33^ esu.

The impact of incident radiation can be assessed by analyzing the HRS data. The results presented in Table 4 were calculated for a laser of frequency λ = 1064 nm, which is most commonly used in experimental measurements. Under gas phase conditions, β_HRS_ yields 68 × 10^–30^ esu. However, when the NTAMP molecule is in aqueous solution, this value changes to 128 × 10^–30^ esu, again indicating a large polarization effect of 88%. This is lower than that obtained for the static case, but still a substantial effect of the solvent on the molecular NLO performance.

We can obtain a qualitative interpretation of the solvent effect on the first hyperpolarizability by considering the two-level model^1^
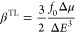
11where Δ*E*, *f*_0_ and Δμ are, respectively, the transition energy, the oscillator strength, and the difference between the permanent dipole moments of the excited state and the ground state. This model establishes a link between the first hyperpolarizability and the so-called crucial electronic transitions. Here, the crucial electronic transition is characterized by small excitation energy, large *f*_0_ and large Δμ. Tables 2 and 3 also report the DFT spectroscopic factors of the two-level model calculated in gas phase and aqueous solution. At the M06-2X level, for instance, the values of β^TL^ for NTAMP are in the increasing order of gas phase (11.4 × 10^–30^ esu) < water (37.3 × 10^–30^ esu), which is in agreement with the order of values of the dominant component, β_*xxx*_: gas phase (43.1 × 10^–30^ esu) < water (132.1 × 10^–30^ esu). Also, we observe that the values of β^TL^ is due to the combination of the oscillator strength and the charge transfer of the crucial transition.

Finally, a relevant aspect pertains to the origins of the first hyperpolarizability. This aspect can be better understood by leveraging the depolarization ratio (DR), which ranges from 1.5 (octupolar) to 9 (dipolar), delineating the nature of these contributions. As per Table 4, all addressed quantum mechanical methods indicate values around DR = 5, regardless of the environment. Furthermore, based on the dipolar (Φ_*J*=1_) and octupolar contributions (Φ_*J*=3_), there exists a balance between these compositions. However, Zhang and collaborators^27^ devised a scale that more accurately classifies NTAMP as a dipolar compound.

### 3.5 Two-Photon Absorption Cross-Section

Table 5 shows the results for the fourth lowest two-photon absorption transitions calculated for the NTAMP molecule in both gas phase and water solvent. The first transition exhibits a negligible cross-section of 0.03 GM at 2.68 eV (462.6 nm), corresponding to the n → π* excitation as predicted by theoretical calculations but not observed in the experiment.

**Table 5:** Two-Photon Absorption Cross-Section Calculated at the CAM-B3LYP/6-311++G(d,p) Level of Theory^a^

			two-photon tensor elements							
environment	transition	energy (eV)	*S*_*xx*_	*S*_*yy*_	*S*_*zz*_	*S*_*xy*_	*S*_*xz*_	*S*_*yz*_	δ_a.u._^2PA^ (a.u.)	δ_GM_^2PA^ (GM)
gas phase	1	2.68	0.0	0.0	–0.0	–0.0	3.3	–0.4	0.02	0.03
	2	2.80	–180.8	–3.5	0.2	41.4	0.0	–0.0	40.7	77.20
water	1	2.56	–435.2	–17.0	0.7	106.6	0.0	–0.0	202	383.15
	2	2.65	–0.0	–0.0	–0.0	0.0	7.4	–0.9	0.08	0.15

a The factors of conversion are 1 au = 1.896788 × 10^–50^ cm^4^ s/photon, and 1 GM = 10^–50^ cm^4^ s/photon.

The highest 2PA value (77.2 GM) is observed at 2.80 eV (442.8 nm), which coincides with the energy of the lowest π → π* transition responsible for the broad absorption band in the visible spectrum. The contributions of *S*_*xx*_ and *S*_*xy*_ to this effect are significant, with values of −180.8 and 41.4 au, respectively. However, considering a recent study that compared the performances of CAM-B3LYP, CC2, and EOM-EE-CCSD methods with different basis sets,^56^ a higher value is expected. According to these findings, the outcomes obtained with CAM-B3LYP are potentially three times lower than those obtained with more advanced techniques like CC2 and EOM-EE-CCSD. Moreover, it is feasible to expect cross-section values of 231.6 GM.

In the aqueous environment, the 2PA cross-section increases considerably. The CAM-B3LYP results associated with the PCM indicate a value of 385.15 GM at 2.56 eV (484.31 nm). That means a strong redshift regarding the gas-phase spectra.

In comparison to other dyes, NTAMP demonstrates enhanced performance. For instance, when considering the pNA molecule, experimental and theoretical methodologies suggest values ranging from 8.04 to 12.17 GM depending on the solvent environment.^57^ First-principle Born–Oppenheimer Molecular Dynamics indicate that these values may be even lower in a water solvent, between 4.4 and 10.3 GM.^58^ In contrast, the predicted values for NTAMP are notably higher than other dyes functionalized to exhibit increased 2PA. This is evident in the case of certain nanosubstituted chalcones,^59^ dibenzylideneacetone, and thiosemicarbazone derivatives.^54,60^

## 4 Conclusions

We carried out a systematic quantum chemical investigation on the effects of solvents on the linear and nonlinear optical properties of NTAMP, a recently synthesized azo compound. Photophysical analysis reveals three notable findings.

The nature of the lowest absorption band, previously assigned as a weak n → π* transition, was investigated. Unlike previous predictions, quantum chemical calculations based on DFT theory show that this band is, in fact, a π → π* state.

The emission spectra of NTAMP were also discussed, highlighting some significant aspects of its photophysics. For example, even in the gas phase, all results indicated a Stokes shift of approximately 125 nm. Furthermore, solute–solvent interactions act to enhance this property. While there are no experimental discussions on the efficiency of this molecule in capturing the emission of light electric current, the reported large Stokes shift and the higher intensity of the lower excitation band suggest potential applications such as sensors, biological probes, light emitting diodes, or solar cells.

Special attention was paid to the first hyperpolarizability, often considered the most relevant nonlinear optical parameter. Although no significant differences were observed between the case of the static field and the frequency-dependent incident light, the solvent enhanced the first hyperpolarizability by approximately 88%, reaching values around 130 × 10^–30^ esu, significantly higher than those reported for urea and *p*-nitroaniline, two standard nonlinear optical compounds. The environment emerged as a key factor in the electronic properties of NTAMP. The simple inclusion of the solvent through the PCM induced a significant bathochromic effect (∼40 nm) in the lowest absorption band π → π*, which was also reflected in the electronic spectra and Stokes shifts.

To conclude this study, a simple analysis of the two-photon absorption cross-section (δ_GM_^2PA^) was performed, indicating superior performance compared with standard dyes such as *p*-nitroaniline and even chalcone molecules.
